# Rethinking the ethical principles of genomic medicine services

**DOI:** 10.1038/s41431-019-0507-1

**Published:** 2019-09-18

**Authors:** Stephanie B. Johnson, Ingrid Slade, Alberto Giubilini, Mackenzie Graham

**Affiliations:** 1 Wellcome Centre for Ethics and Humanities, University of Oxford, Oxford, UK; 2 Ethox Centre, University of Oxford, Oxford, UK; 3 Uehiro Centre for Practical Ethics, University of Oxford, Oxford, UK

**Keywords:** Ethics, Public health

## Abstract

Clinical genome and exome sequencing is currently used in only a small fraction of patients, yet large scale genomic initiatives are becoming more embedded in clinical services. This paper examines the ethical principles that should guide regulatory processes regarding consent and data sharing in this context. We argue that a genomic dataset administered by the health system carries substantial societal benefits, and that the collective nature of this initiative means that at least those patients who benefit from genome sequencing have an ethical obligation to share their health information. This obligation is grounded in considerations of fairness. Furthermore, we argue that the use of genomic data for the advancement of medical knowledge should be permitted without explicit consent and that international and other bodies should be granted access to these data, provided certain conditions are satisfied.

## Introduction

In October 2018, the United Kingdom’s National Health Service (NHS) introduced whole genome sequencing into routine NHS care, as part of the world’s first ‘Genomic Medicine Service’, with the ambition to sequence 5 million genomes in the next 5 years. Other countries, including France and Canada, are at various stages of establishing similar publicly funded genomic healthcare services [1].

It is hoped that establishing genomic services of the scale initiated within the NHS will provide important benefits. Clinical genome and exome sequencing is currently used in only a small fraction of patients, principally for the diagnosis of suspected Mendelian conditions and for targeting cancer treatments [2]. Expanding the use of whole genome sequencing into routine care has the potential to expand diagnoses outside of cancer and rare diseases, including prenatal testing. In the future, genomics may be used to determine optimal drug therapy and dose given a person’s metabolic response; to allow for more accurate prediction of individual susceptibility to disease; and to increase understanding of its underlying cause. Within this context, there are many benefits to collection, use and storage of genomic data for individuals and society at large.

In addition to the potential benefits, a clinical genomics service will also raise important ethical, social and legal challenges. One key challenge is the hybrid nature of genomic medicine initiatives. Genomic medicine aims to both provide patients with a clinical diagnosis or personalised/targeted treatment and embed knowledge generation into healthcare practice (i.e. data produced by clinical tests enters a research database). The deliberate integration of research and clinical practice, which have historically been kept distinct [3], raises questions about what ethical principles should govern this practice; those of clinical care or those of research? Increased collection and use of personal medical and biological information such as that required by genomics also raises issues of privacy and security, and challenges the adequacy of traditional conceptions of informed consent [3]. Importantly, as an effective genomic service requires a large and diverse database of genetic information, new questions will arise. For example, what are the ethical obligations of patients to contribute, share and link their genomic information for the mutual benefit of themselves, and others?

This paper aims to address some of these concerns and argues in support of the routine collection and linkage of individual genomic data within publicly funded healthcare systems. We argue that public genomic datasets carry substantial societal benefits, and that the collective nature of these initiatives means that those patients who benefit from genome sequencing have an ethical obligation to share their health information, an obligation grounded in considerations of fairness. We argue that in order to maximise the benefits of genomic services, the storage and use of genomic data for the advancement of medical knowledge should be permitted without explicit and specific consent, and that international and other bodies should be granted access to these data, provided certain conditions are satisfied (Box 1). While the considerations here are largely specific to the NHS, they may form an exemplar for further international work.

Box 1 proposed conditions to routine collection, storage and use of data

- Genomic dataset should be appropriately secure and consist of de-identified data, with genomic and phenotypic data not linked to personal information
- Stringent data protection policies and legislation should be a requirement of genomic services
- Risk to data security should be balanced against the potential benefits to patients
- Terms of agreement regarding access to data are required
- Understanding social and legal risks, and ongoing monitoring of the legal environment are required
- The benefits and acceptability of public–private genomic services should be established
- Inequalities in access to the benefits of genomic services must be addressed
- Practices related to data collection, storage and use must be acceptable to and command the trust of the public

## The collective nature of genomic datasets

Achieving the benefits of genomic medicine is dependent upon ongoing collection and storage of large amounts of genomic and phenotypic data [4, 5]. This is because understanding genetic variation and its association with common and complex disorders on a genome-wide scale requires large sample sizes to achieve sufficient power [6]. When a genomic test is carried out, the discovery of rare changes in the DNA can only be understood by comparing an individual’s result to that of many thousands of others. For example, patients with cancer can have both their somatic and constitutional genome sequenced (and re-sequenced over time), in order to determine which kind of chemotherapy is most likely to treat their cancer, based on what has worked in other similar patients. A requirement of an effective clinical genomic service, therefore, is to have an established centralised dataset, containing genotypic and phenotypic information from previous tests.

This has three key implications. First, an effective genomic service can only be realised through the joint contribution of a great number of the population. Second, the larger the dataset, the greater the potential benefit to current and future patients. Third, individual benefit can only be realised through contribution from the collective. In other words, one individual cannot interpret their genomic test without ongoing systematic collection of data from other persons. This is important because it means that genomic datasets, such as the one established by the NHS can reasonably be conceived as a public resource. Health datasets can be considered a public resource when: (1) they include data regarding many, if not all, citizens, (2) they hold value for the public (i.e. future individuals and families) and (3) public expenditure is necessary for generating the data, which creates a public claim on it [7]. An NHS genomic dataset meets all three conditions.

Our suggestion is that there is an ethical obligation to manage public resources in a way that maximises public benefit [7] and that therefore, future approaches to the regulation and governance of clinical datasets should be grounded in an ethical duty to maximise the potential social benefits of data. Furthermore, we argue (below) that there is an ethical obligation to manage data in a way that is fair, provided that does not unduly burden individuals. This provides strong grounds for requiring patients who undergo clinical genomic testing to allow their genomic data to be incorporated into a genomic dataset.

## Fairness as a moral imperative for publicly funded genomic datasets

A critical aspect of maximizing the public benefit of a genomic dataset is ensuring an adequate supply of diverse, high-quality genomic data. In this sense, aggregate individual contributions are required to ensure that the potential benefits of these databases are realized. We argue that the benefits and burdens of this collective responsibility should be allocated fairly. While fairness does not require that everyone contributes or receives the same [8], in the case of contributing to a genomic database, we argue that everyone has a duty to share their genomic information, but that this requirement is stronger for patients who stand to directly benefit from such a database.

Fairness may be conceived of and defined in a number of ways; for our purposes, we will focus on three: as a matter of reciprocity; as a matter of fulfilling one’s share of a collective obligation; and as a way of avoiding free riding.

The concept of reciprocity can be understood in a direct, and an indirect sense. Direct reciprocity entails an obligation to give something in return to a third party, when one benefits from a good that the third party provides or to which the third party contributes. ‘Indirect reciprocity’ [9, 10] entails an obligation to make a contribution to a system without knowing whether what she will receive in return will be of the same kind that she has given, or whether she will even ever need it. Direct reciprocity obligates only those patients who benefit from the database to provide something in return for this benefit, at least when doing so does not pose an unreasonable burden on them. Conversely, indirect reciprocity implies that everyone has a duty to contribute to the database even if they do not stand to benefit, or cannot be certain they stand to benefit. While not all patients will necessarily benefit from the existence of a genomic database (depending on their illness), access to the database will be available for the treatment of all patients. Accordingly, all potential beneficiaries of the database have an obligation to contribute, according to the requirements of indirect reciprocity.

Second, fairness requires contributing one’s ‘fair-share’ to a collective good. In many cases of achieving a collective good, the overall benefit arising from the contribution of one individual is negligible. Thus, the obligation to contribute cannot be justified by the impact that one’s contribution will make. For example, whether Jane contributes her genomic data to the database will have minimal impact on the overall efficacy of the database, and thus, its overall benefit to society. Jane does not fail to benefit anyone and does not harm anyone by not contributing. However, fairness requires that the burdens of a collective responsibility—such as maintaining a genomic database—are equally distributed among the relevant collective [11]. This obligation to ‘do one’s share’ has been defended in a range of contexts in public health, such as herd immunity through vaccination [12, 13].

Determining what constitutes one’s ‘fair-share’ of a collective obligation is complex. On the one hand, strict equality would imply that everyone ought to contribute an equal share, regardless of their circumstances. On the other hand, one might think that we should demand less from people who have less in the first place than from privileged and well-resourced people [8]. Against this background, it could be argued that it would be unfair to expect data donation from sick patients, because they are already burdened by ill health. If contributing one’s genomic data were significantly more burdensome for a sick patient than for a healthy person, this would weaken the obligation of the patient to contribute. However, as we argue below, the burdens of contributions are low, and largely similar across patients. Moreover, systematic exclusion of particular subgroups of patients (e.g. by disease or demographic group) from data collection and storage would lead to reduced utility of the data and poorer health outcomes for other members of those subgroups. For example, the long-time exclusion of pregnant women from randomized clinical trials has led to a dearth of reliable evidence for the safe dosage and efficacy of drugs for this population. Excluding certain groups from a genomic database would result in members of these groups receiving similarly inferior treatment. Such a policy risks introducing an additional source of injustice to the health system. Whether patients with rare diseases, or members of minority groups, have a special obligation to contribute their genomic data in order to reduce potential injustice, is a question which we will not consider in this paper. Rather, we argue that insofar as one’s ill health does not exacerbate the burdens of contributing one’s genomic data, those burdened by ill health have at least as strong an obligation to contribute as healthy citizens.

Finally, a genomic database carries an opportunity for ‘free-riding’, that is, for individually benefitting from a collective good without also taking on the burdens of contributing to it. This is a classic problem of collective action that is found in numerous contexts (climate change, herd immunity, taxation). Free riding is normally taken to be unfair. Without external restrictions requiring contribution to the database, anyone who became sick and required a genomic test could benefit from the genetic database by gaining the fullest understanding of their genomic information, and therefore optimal care, without having to make their genetic information available for inclusion in the database.

Given the requirements of fairness outlined above, justice would be realised if at least those patients having a genomic test contribute their data to a genomic dataset. While the second conception of fairness—contributing ones’ fair share to the collective good—would demand that every individual disclosed their genetic information, the first and third conception of fairness demand that at least those who directly benefit from a genomic dataset contribute to its continuous learning. Whether moral obligations of fairness are strong enough to justify coercive public policies is contested in many areas of healthcare relating to collectives action problems. These include vaccination and organ donation. Our suggestion is that patients who undergo clinical genomic testing *should* incorporate their data into a genomic dataset; and there are ethical grounds for modelling policy on the basis of this and other ethical assumptions (see Prainsack and Buyx, modelling biobanks on the solidarity assumption) [14].

## Rethinking ethics frameworks

We have argued that genomic datasets are a public resource that carry large societal benefits, and that they should be managed in a way that maximises those benefits, and is consistent with the requirements of fairness. This means states or governing bodies have an ethical obligation to maximise contributions to the dataset and to maximise the interoperability of the databases, and grant access to authenticated researchers internationally [15]. Patients who undergo clinical genomic testing should allow their data to be incorporated into a genomic dataset.

We argue that it is ethically permissible to collect and store a patient’s genomic data, without their explicit consent, for the purposes of developing a genomic dataset and as a means of maximising contribution to the dataset. When a patient is given the option of receiving a genomic sequence through the NHS, they should be informed that their data will subsequently be used to supplement the existing database, as part of the commitment of the NHS to maximize the public good. The patient should also be made aware of the potential future uses of their data, but their explicit informed consent for these uses is not required. In addition, infrastructure should be provided to support the systematic collection and storage of population datasets. Genomic datasets should be expanded to include existing NHS patient databases for greater linkage and collection of genomic and phenotypic data, and access to appropriately secured data should be provided to authorised users internationally. In return, the obligation of the governing body overseeing the development and maintenance of the dataset (i.e. the NHS), is to ensure that individuals contributing their data receive adequate protection of their interests. Indeed, part of maximizing the potential benefits of a public good like a genomic dataset is minimizing the potential for harm. As we discuss in the next section, the potential harms to individuals providing their genomic data are minimal, and can be ameliorated further by rigorous data security measures.

## Addressing criticisms

### Genetics as a special case

Historically, certain types of information have been regarded as requiring particularly stringent privacy protection, or explicit and specific consent processes (e.g. testing for HIV). It has been proposed that genomic data requires similar special considerations [16, 17]. Proponents of this view claim that genetic information is uniquely powerful and personal, and therefore deserving of unique protection[16]. Various arguments have been offered in support of this view: genetic information is fundamentally important to some understanding of personal identity; access to and control of genetic information make it possible for others to have power over a person’s life and to predict their future; genetic information provides information about an individual’s family members; genetic information is uniquely identifying; and the ease with which DNA testing can be carried out means it could be used surreptitiously [18].

This form of ‘genetic exceptionalism’, however, has also been contested [19]. First, on any plausible understanding of personal identity, many factors other than genes determine who a person ‘is’ [20]. Moreover, research in population genomics continues to demonstrate that genetic risk factors offer only probabilistic estimates of future disease, undermining the claim that genes are determinative of current or future health [21]. In addition, many diseases cannot be neatly classified as genetic or non-genetic, and genetic information can be gleaned from sources other than DNA [21]. Non-genetic forms of clinical testing, such as blood pressure measurement, studying familial patterns of disease, chloride tests on perspiration to test for cystic fibrosis, and cholesterol tests, can also be highly predictive of genetically based disease [22].

While genetic information can provide information regarding close relatives, some have argued that precisely because genetic code is shared, genetic information such as a familial predisposition cannot be considered ‘personal and sensitive’, given that it is not identifying or unique to any one person [23]. Furthermore, (as above) genetic data is not distinctive in holding importance for families.

Lastly, although genomic data is strictly speaking unique, it is not intrinsically identifiable. Rather, it must be matched to a particular patient through other identifying information. A database of ‘genetic code’ is only identifiable if matched to a patient through separate linking data, much as knowing a person-unique fact such as a social security number permits identification only if it can be traced to the person through some other source [24].

Genetic information collected in a public genomic dataset like the one described in this paper are not exceptional. The data is no more uniquely personal, sensitive or wholly different from other data collected for medical purposes, and we must apply the same rigorous principles as we would for other medical information, but no more [25].With this in mind, although data collected for health purposes cannot generally be used for secondary purposes (purposes that are different from the ones for which data were collected in the first place) without explicit consent, many exceptions are made to the consent requirement for secondary use. These include cases where gaining consent would be impractical or would impede the scientific validity of the study, and where the study addresses important health questions and poses minimal harm to participants [7, 26–29]. Ethical exceptions have also been made for public health surveillance research, such as cancer and notifiable disease registries. Within the NHS, data regarding patients’ interactions with secondary care services is recorded on statutorily defined datasets. Hospital episode statistics—including over 125 million admitted patient, outpatient and accident and emergency records each year—are also routinely collected. These datasets are normally taken to be ethically permissible, because of the wider collective benefits and minimal risk of harm to individuals. Therefore, given our arguments against genetic exceptionalism, a NHS genomic dataset would not be ethically distinct from currently accepted practices regarding the collection of personal information for public benefit, provided it was appropriately secure and consisted of de-identified data, with genomic and phenotypic data not linked to personal information. Such a database could operate under similar consent and privacy principles to healthcare data that is currently routinely collected.

### Privacy and data security

Concerns may be raised that accidental data release, or criminal offences including hacking or data theft could result in serious violations of privacy [17]. In practice, identification of an individual through knowledge of their genetic variant(s) is difficult, and re-identification would require an intimate knowledge of the individual’s genotype or phenotype together with some information to trace that genotype/phenotype to a specific person [30]. Joly and Knoppers [30] propose that, in practice, only an individual patient or their clinician would easily be able to re-identify themselves from a specific variant. In addition, a variety of methods that could be used to reduce the identifiability of data have been proposed. These include limiting the proportion of genomic data released, statistically degrading data or sequestering identifiers via key coding (reversibly de-identifying) [24]. Lowrance & Collins [24] claim that controlled-access models can keep the risk of identifying individuals low.

While stringent data protection policies and legislation should be a requirement of genomic services, it should also be acknowledged that this can only ameliorate risk, as developments in informatics show that the guarantee of absolute privacy and confidentiality is not a promise that the medical world can deliver any longer [31]. Similar risks are normally considered acceptable for other types of sensitive personal data, such as financial data. The benefits associated with the current collection of healthcare data (i.e. for resource allocation and quality improvement) justify the small potential for harm associated with a breach of data security. Similarly, with respect to genomic datatsets, it will be important that risk to data security is balanced against the potential benefits to patients. Provided the appropriate steps are taken to minimize the potential for a breach of data security with respect to genomic data, the considerable benefits to patients is likely to significantly outweigh this risk of potential harm.

### Access to data

Concerns might also be raised about risks of inappropriate use of data and resulting legal or financial ramifications; stigmatization; and/or discrimination for insurance, employment, promotion or loans. Terms of agreement regarding access to data are required. The UK, for example, has a moratorium on insurance and genetics. Currently, the only predictive genetic test results that can be asked for and used by insurers is when an individual has a predictive genetic test result for Huntington’s disease and they are applying for over £500,000 of life insurance, and this arrangement is likely to continue [32]. In fact, early fears relating to genetic discrimination and the impact of genetic data on insurance premiums have proven to be largely unfounded in the UK and many other countries (5, 30, [5]). Nonetheless, understanding social and legal risks, and ongoing monitoring of the legal environment, will be an essential part of establishing and maintaining public willingness in contributing to a genomic database.

Further to this, an explicit goal of Genomics England is to provide academic and/or commercial researchers with access to patient data [33]. Surveys have shown that members of the public are willing to share healthcare data within the healthcare service and for research when the aim is for the public good, but that concern is raised when healthcare data is to be shared with companies for profit-making purposes [34, 35]. It seems likely, however, that genomic medicine services will eventually involve private companies. Genomic data will be a valuable resource, and ensuring the optimisation of the benefits that can come from integration of such data will require collaboration with industry, including major UK centres accomplished in computational biology and outcomes data [34]. Managing relationships in a way that maintains public trust and provides benefit sharing for the NHS will be essential. As there is a necessity for the dataset to be regularly refreshed, this is unlikely to be a one-time exercise. Proposed models include pay for access (with money going directly to the NHS), or the NHS receiving a percentage of profits from work using their data, or the NHS having shares in the private company. None of these solutions propose to financially compensate the individual whose data is used, but rather aim to benefit the collective. We suggest that further empirical work examining the benefits and acceptability of public–private genomic services is urgently required.

### Legal and regulatory context

Concerns may be raised that data protection regulations, such as the new European General Data Protection Regulation (GDPR), may present significant practical barriers to the routine collection and storage of data without explicit consent. However, existing exemplars suggest that regulatory and legal frameworks do not pose significant barriers to the responsible collection, storageand use of genomic data, and that a genomic database as proposed would be compliant. According to GDPR requirements for processing personal data under the scientific research exemption, pseudonymized data within a controlled-access network and with approved users—under which the further use of genetic data for scientific research purposes would be included—obtaining explicit consent is not required [36]. Two existing public health databases currently operate under this principle. ClinVar and DECIPHER (the Database of Genomic Variation and Phenotype in Humans Using Ensembl Resources) are two major public databases that are frequently used by laboratories for data sharing [37]. Consistent with GDPR requirements, ClinVar does not require explicit consent for sharing de-identified variant-level information obtained by laboratories during the course of fee-for-service clinical testing [38]. However, these consent requirements may change when sharing ‘more specific individual-level information, such as the distinct phenotypes of each individual observed in a particular laboratory’s experience with a variant’ [38]. Similarly, in a guidance document provided by DECIPHER, explicit consent for data sharing is required only when data are shared open access [37]. Furthermore, in the UK, the European GDPR mandates that stored data are ‘adequate, relevant and limited to what is necessary in relation to the purposes for which they are processed’ [39]. Wright et al. [5] suggest that proportionality in genetic data sharing, that balances the depth of data shared with the breadth of sharing would be consistent with data privacy laws such as UK-GDPR [5, 40].

### An equitable service

Research suggests that disparities exist in access to clinical genetic services, and in the efficacy of those services. African-American women, for example, have been shown to have poorer access to *BRCA1* genetic testing than white women [41, 42]. Likewise, studies have shown that patients of African and Asian ancestry are currently more likely than those of European ancestry to receive ambiguous genetic test results after exome sequencing, or be told that they have variants of unknown significance [42]. Tackling inequalities such as these is important both because a lack of ethnic diversity in genomic medicine substantially decreases the capacity for social benefit (for both minorities and non-minorities) [43–47], and because if all patients are expected to contribute data to a genomic service, reciprocity suggests that all patients should receive equal benefit. Community engagement, improved access to translators and language/culturally sensitive material, as well as strategic changes in research requests and service design to ensure adequate representation of minority groups, have been suggested as possible means of addressing these inequalities [44].

### Public trust

We have outlined many circumstances, in which risks must be carefully examined and considered (see Box 1 for summary). An important step in designing and implementing ethical and governance frameworks which balance these risks will be to ensure that any practices would be acceptable to and could command the trust of the public. While protection of identifiability seems obligatory, there remains important work to be done surrounding the public views on terms of agreement regarding access and use of data, the role of private industry and the concerns of specific cultural and ethnic groups.

## Conclusion

We have argued for some specific approaches to regulation and consent to use of genomic data in public healthcare systems. This includes that the routine collection, storage and linkage of genomic data to be held within the healthcare service records is ethically permissible without specific consent, and that authorised access to data should be encouraged. More importantly, we have made a case that the integration of genomic medicine in healthcare presents an opportunity to re-evaluate and design ethics frameworks, in ways that are relevant to modern healthcare services. Ethics frameworks will need to address issues of consent and regulation arising from the use of these clinical datasets, but the interests of publicly funded medical services and privacy protection must be weighed against each other, rather than defaulting to a position that emphasises individual privacy and autonomy. Ethics frameworks should aim to provide services that maximise social benefit, and encourage the satisfaction of collective obligations in a way that is fair and equitable.
